# Improved curdlan production with discarded bottom parts of Asparagus spear

**DOI:** 10.1186/s12934-017-0671-3

**Published:** 2017-04-07

**Authors:** Rex Frimpong Anane, Huifang Sun, Lamei Zhao, Le Wang, Chun Lin, Zichao Mao

**Affiliations:** 1 College of Agronomy and Biotechnology, Yunnan Agricultural University, Kunming, 650201 China; 2 Institute of the Improvement and Utilization of Characteristic Resource Plants, Yunnan Agricultural University, Kunming, 650201 China

**Keywords:** *Agrobacterium* sp. ATCC 31749, Asparagus, Curdlan, Flavonoids, Saponins

## Abstract

**Background:**

This work evaluated the improvement of curdlan production of *Agrobacterium* sp. ATCC 31749 by using culture medium containing juice of discarded bottom part of green Asparagus spear (MJDA). Curdlan production was carried out using *Agrobacterium* sp. ATCC 31749 in flasks with different volumes of MJDA and its non-juice-adding control (CK) incubated in shaker at 30 °C, 200 rpm rotation for 168 h.

**Results:**

All MJDA media increased *Agrobacterium* sp. ATCC 31749 cell mass and enhanced the cells’ ability to utilise sucrose, the carbon source for curdlan biosynthesis, and thereby produced higher concentration of curdlan than CK which is used for commercial production of curdlan. After 168 h of fermentation, 10% MJDA produced 40.2 g/l of curdlan whiles CK produced 21.1 g/l. Curdlan production was increased by 90.4% higher in 10% MJDA than CK. Curdlan produced by 10% MJDA contains 1.2 and 1.5 µg/ml of Asparagus flavonoids and saponins respectively as additives which have wide range of health benefits. The mass of sucrose needed to produce 1.0 g curdlan by *Agrobacterium* sp. ATCC 31749 in CK is 1.7-fold more than in 10% MJDA.

**Conclusion:**

The results strongly revealed that 5–10% MJDA is a good curdlan fermentation media which increase curdlan production yield with cheaper cost of production and simultaneously reduce environmental waste resulting from the large scaled discarded bottom parts of green Asparagus spear during Asparagus production.

## Background

Curdlan is a water-insoluble exopolysaccharide produced by fermentation process by the *Agrobacterium* sp. ATCC 31749 [1]. It is composed of glucose units of linear β-(1,3)-glycosidic linkages and its biosynthesis involves four genes *crdA*, *crdS*, *crdC* and *crdR* [2, 3]. Its ability to form a thermo-reversible and irreversible gel by heating its aqueous suspension at 55 and 80 °C respectively followed by subsequent cooling, makes it possible to form gels with different strengths [4]. Due to this thermal gelling unique property, it is extensively applied in food and pharmaceutical industries. Curdlan has been reported to be a useful additive for a variety of food products, such as for yogurt stability improvement [5], noodles, sauces, frozen foods and packaged meats for texture improvement and stabilizing ability [6]. Curdlan and its derivatives have also been used as drug delivery vehicles [7] and for sustained release of drugs [8]. Curdlan sulfates has been shown to exhibit high anti-AIDS activity [9, 10] and strong inhibitory action on blood coagulation and hence can be used for the treatment of thrombosis [11]. Curdlan also efficiently modulates both innate and adaptive immune responses by acting on immune receptors such as Dectin-1 and cells such as macrophages, neutrophils, monocytes, NK cells and dendritic cells [12, 13]. It has been reported that curdlan together with activated carbon adsorbents, also has heavy metal removal ability [14]. These and many other reports suggest a huge demand for curdlan application in biomedicine.

Beside the necessity for efficient production process to meet the growing demand for curdlan, the cost of production is also a problem for large scale commercial curdlan producers [15, 16], since the cost of curdlan mainly depends on the raw materials, carbon and nitrogen sources [17]. Sucrose is commonly used as carbon source for the production of curdlan by *Agrobacterium* sp. ATCC 31749 and this increases the production cost, since sucrose, glucose, fructose and maltose which are the major raw materials for commercial polysaccharide production are very expensive. Therefore, research is being carried out on the use of alternate carbohydrate-rich sources, such as molasses, sugarcane juice, jaggery [18], starch hydrolysates and the like, which are more economical compared to the currently pure sugars being used [19]. Portilho et al. [20] produced succinoglycan and curdlan by two *Agrobacterium* sp. strains (ATCC 31749 and IFO 13140) from alternative fermentation media using glucose from maize and cassava starch hydrolysate, and maltose from maize starch hydrolysate as the main carbon source. Other literatures reveal that cordgrass hydrolysate and condensed corn distiller soluble have been applied as effective carbon sources for curdlan production [21, 22]. Another study, indicated the possibility of using date palm juice by-products as the main carbon source for curdlan production by *Rhizobium radiobacter* ATCC 6466 [17, 23]. Coconut water, an environmental friendly and a natural carbon source, has been exploited for the production of exopolysaccharides such as curdlan by *Agrobacterium* sp. CFR-24 [19].

Asparagus is a multipurpose vegetable that has both nutritious and medicinal functions [24] such as anti-inflammatory activity [25]. Almost one third of the total length of the edible part of Asparagus (bottom and underground part of each spear) is discarded, which represents an important loss for the Asparagus producers. These discarded by-products have similar composition to that of the edible part of the plant [26] hence a good source of minerals, vitamins, flavonoids, saponins, proteins and carbohydrates [27]. To our knowledge, there is no available literature on the use of *Asparagus* juice for formulation of culture media for curdlan production. This research therefore aim to produce a novel culture medium, containing juice of discarded underground and bottom part of Asparagus spear (MJDA), to improve curdlan production with a lesser cost of production.

## Methods

### Bacteria strain and chemicals

The bacteria strain *Agrobacterium* sp. ATCC 31749 used for curdlan production was obtained from the American Type Culture Collection (ATCC, Manassas, VA, USA). It was stored in a complex medium in −80 °C refrigerator in a laboratory in the Biochemistry and Biotechnology Department of the College of Agronomy and Biotechnology, Yunnan Agricultural University, Kunming, China. All chemicals used in this study were obtained from Sigma Aldrich, USA.

### Preparing the juice of discarded Asparagus (JDA)

Discarded part of Asparagus which were obtained from Asparagus growing base of Cheng Zhang Agricultural Development Co Ltd. in Kunming, Yunnan of China were washed and packaged into bags of 1 kg each. Prior to grinding for juice or storage at −20 °C, the *Asparagus* materials were air dried for about 1 h. The extraction of the juice was performed by grinding 1 kg of the discarded Asparagus part and the juice was squeezed out. The obtained juice was centrifuged (9000×*g*, 20 min, 25 °C). The resulting supernatant was further filtered three times with Whatman no. 1 filter paper and the filtrated solution was the juice of discarded Asparagus (JDA).

### Culture medium (MJDA)

The LB agar medium for stock cultivation contained (w/v): 1% tryptone, 0.5% yeast extract, 1.5% agar, and 1% NaCl, pH 7.0. The seed culture LB medium consisted of (w/v): 1% tryptone, 0.5% yeast extract, and 1% NaCl, pH 7.0. The curdlan fermentation medium (CFM) for flask cultures also contained (in g/l): sucrose 50.0, citric acid monohydrate 5.0, yeast extract 0.1, K_2_HPO_4_ 2.7, KH_2_PO_4_ 1.7, MgSO_4_ 0. 05, NH_4_Cl 0. 07, plus 1% minerals solution (1 g FeCl_3_, 1 g MnCl_2_, 1 g NaCl, 1 g CaCl_2_ in 100 ml d∙H_2_O) [16] was used as the control medium (CK). The 5% JDA, 10% JDA, 15% JDA and 30% JDA was mixed with appropriate medium contained (in g/l): sucrose 50.0, citric acid monohydrate 5.0, yeast extract 0.1, K_2_HPO_4_ 2.7, KH_2_PO_4_ 1.7, NH_4_Cl 0.007% to obtain their respective MJDA. No minerals was added to MJDA. All media were sterilized by autoclaving (Shanghai Boxun Ind.) at 121 °C for 15 min unless otherwise stated.

### Seed culture

The −80 °C *Agrobacterium* sp. ATCC31749 stocks were streaked on LB Agar to obtain separated colonies of the strain. A single colony of the fresh LB agar culture was inoculated into a 5 ml LB broth in 50 ml Erlenmeyer flask and incubated in a rotary shaker (Tensuc TS-2102 shaker incubator, Beijing, China) at 30 °C and 200 rpm for 12–24 h. 1 ml of this culture was then inoculated into 100 ml LB broth in 500 ml Erlenmeyer flask and incubated in a rotary shaker at 30 °C and 200 rpm for 24 h as the seed culture.

### Optimization of MJDA fermentation media

The MJDA fermentation media were prepared with 5, 10, 15, 30, 40, 50, 60, 80 and 100% MJDA (in 100 ml d∙H_2_O, no addition of sucrose). The Erlenmeyer flasks containing 100 ml each of these MJDA media were inoculated with 5 ml of the seed culture and incubated on a rotary shaker (200 rpm for 168 h at 30 °C) respectively. The fermented broth (MJDA) were analysed for curdlan content in g/l to determine the optimum JDA percentage for highest curdlan production. The obtained optimum JDA percentages were then used for the present study.

### Production of curdlan

Fermentative production of curdlan was carried out with *Agrobacterium* sp. ATCC 31749 in a rotary shaker (30 °C, 200 rpm). 5 ml of the seed culture was inoculated into 100 ml of each of the optimum MJDA fermentation media that yielded highest curdlan production (in 500 ml Erlenmeyer flasks). The flasks were incubated in a rotary shaker at 200 rpm, 30 °C for 168 h [28]. Fed-batch culture (two stage) fermentation procedures were conducted in 500 ml Erlenmeyer flasks. 5 g sucrose was added at 0 and 48 h each. The pH was controlled at 7.0 during cell growth stage (0–24 h) and at 5.5–6.0 pH for curdlan production stage (24–168 h) by addition of 1 M NaOH or 2 M HCl solutions [29] respectively. The flasks were removed from the rotary shaker at 48, 96 and 168 h, and fermented broth samples were taken and analysed for various parameters. All procedures were taken in sterile environment.

### Determination and quantification of curdlan and biomass yield

The amount of curdlan and biomass produced by *Agrobacterium* sp. ATCC 31749 were determined by the dry weight method [30] with slight modifications. A 20 ml sample of the fermented broth was removed from each flask and centrifuged at 10,000×*g* for 10 min at 25 °C (HC 3018R, Zonkia Scientific Instruments, Hefei, China). The supernatant was removed and used for various media composition (sucrose, protein, flavonoids and saponin) analysis, and 20 ml of 1 M NaOH solution was added to the precipitate and incubated at 25 °C for 60 min to solubilize the curdlan. Afterwards, the mixture was centrifuged at 9000×*g* for 10 min at 25 °C and the resulting supernatant was collected. The supernatant was used to determine curdlan production whiles the cell pellet was used to determine the cell mass. The curdlan in the collected cell-free supernatant was precipitated by adding 2 M HCl to the supernatant until the solution pH reached 6.5. Recovery of the precipitated curdlan was performed by centrifugation at 10,000×*g* for 10 min at 4 °C and the product was washed with double distilled H_2_O to remove the salts. The precipitated curdlan was collected on a pre-weighed Whatman no. 1 filter paper. The amount of curdlan was measured by weighing after drying at 80 °C to constant weight (gl^−1^).

### Soluble sugar determination

The sucrose concentration was measured using the anthrone-sulfuric acid method [31]. 2 ml aliquot supernatant of fermented MJDA was added to 20 ml test tube. 5 ml 0.2% anthrone-sulfuric acid was added to the flask containing the sample. The solution was allowed to cool down at room temperature for 10 min. The absorbance was measured​ against blank at 620 nm. The calibration curve was prepared exactly by the same protocol by using 0.01% sucrose solution (0–100 µg/ml).

### Total flavonoids determination

Total flavonoids content was determined by aluminum chloride colorimetric method as described by [32]. 1 ml aliquot supernatant of fermented MJDA was added to 25 ml test tube containing 4 ml of distilled water. 0.3 ml 5% NaNO_2_ was added to the dilution. After 5 min, 0.3 ml of 10% AlCl_3_ was added. After 6 min of incubation at room temperature, 2 ml of 1 M NaOH was added. The total volume was made up to 10 ml with distilled water. Absorbance was read at 510 nm against reagent blank with a spectrophotometer. The standard curve was prepared by catechin solutions of 2–8 µg/ml. Based on the measured absorbance, the concentration of total flavonoids was then calculated (µg/ml).

### Total saponins determination

Total saponins was determined by vanillin–ethanol assay [33]. 1 ml aliquot supernatant of  fermented MJDA was dissolved in 5 ml 80% methanol. 2 ml of 5% vanillin–ethanol was added and mixed well. 2 ml of 72% H_2_SO_4_ solution was added and mixed well. The solution was heated on water bath at 60 °C for 10 min. Absorbance was measured at 544 nm against reagent blank. The calibration curve was prepared by Diosgenins solution (0–80 µl/ml).

### Total proteins determination

The spectrophotometric method of Bradford was used to determine total proteins [34]. 1 ml aliquot supernatant of fermented MJDA was dissolved in 1 ml Bradford reagent and mixed well. The solution was incubated at room temperature for 10 min. Absorbance was measured at 595 nm against reagent blank. The standard was prepared by BSA (0–120 µg/ml).

### Saponins and flavonoids determination in curdlan

One millilitre freshly extracted curdlan was dissolved in 10 ml distilled water in a 20 ml test tube. The suspension was placed in a water bath at 70 °C for 15 min to dissolve the curdlan. The concentration of saponins and flavonoids in curdlan was determined by their respective spectrophotometric methods.

## Data analysis

All data analyses were conducted in triplicate for each parameter and the efficiency was obtained by the average of the triplicates. The results obtained were entered into GraphPad Prism 5 software (7825 Fay Avenue, Suite 230 La Jolla, CA 92037 USA) for one way analysis of variance, ANOVA, and Tukey tests analysis at a 5% significance level.

## Results

### Optimization of MJDA fermentation media

Carbon source is important for curdlan synthesis since. During microbial fermentation, carbon source act as the major component for the production of cellular materials as well as source of energy [35]. Literature reports that sucrose is the most suitable carbon source for curdlan production by *Agrobacterium* sp. ATCC 31749 [15]. The present study aimed to use JDA as a supplementary source of partial carbon and energy to sucrose. The effects of different concentrations (5–100%) of MJDA on curdlan production were analysed and it was noted that 5–15% MJDA produced highest amount of curdlan compared to the other concentrations of MJDA (Table 1). In order to further investigate the effect of MJDA on curdlan production and its efficiency and co-effectiveness with sucrose, the optimal concentrations (5, 10 and 15% MJDA) were then selected for the present study with the control medium (CK).

**Table 1:** Optimization of MJDA culture media

2MJDA (%)	Cell mass (g/l)	Weight of curdlan produced (g/l)
5	3.0186 ± 0.1673	5.2933 ± 0.7328
10	3.2894 ± 0.1243	6.3867 ± 1.0134
15	3.1576 ± 0.0918	4.8463 ± 0.5916
30	2.9812 ± 0.1672	4.0265 ± 0.6009
40	2.8295 ± 0.2619	2.5133 ± 0.3428
50	2.7831 ± 0.1967	2.0300 ± 0.4117
60	2.4500 ± 0.3057	1.8933 ± 0.6306
80	2.5134 ± 0.0986	1.5291 ± 0.5937
100	2.6347 ± 0.2618	1.4642 ± 0.3986

### Effect of MJDA on curdlan production

**Fig. 1:**
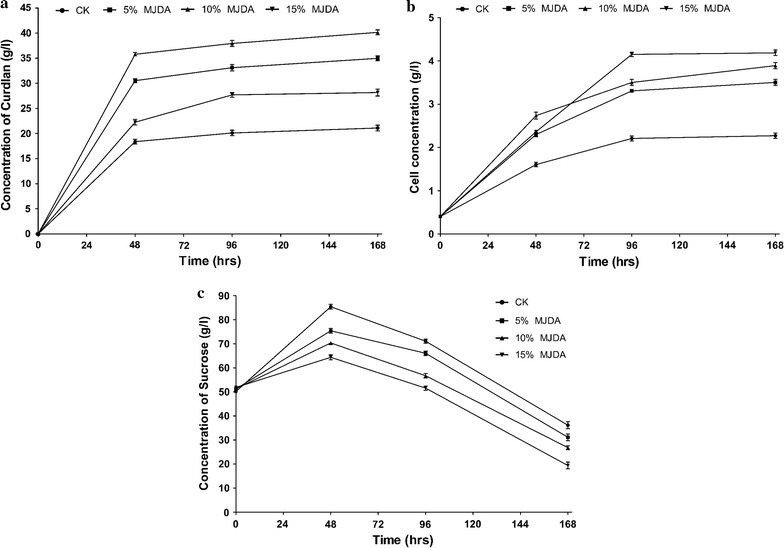
**a** Concentration of curdlan produced after 168 h. All MJDA media produced higher mass of curdlan compared to CK. Curdlan production increased with respect to time but the increase from 96 to 168 h was minimal. **b** Cell growth from 0 to 168 h. Concentration of *Agrobacterium* sp. ATCC 31749 cells in MJDA media were higher than CK. This contributed to the higher masses of curdlan produced in all MJDA. **c** Consumption of sucrose by *Agrobacterium* sp. ATCC 31749 cells. The rate and concentration of sucrose utilized in all MJDA were higher than CK. MJDA media enhanced the cells ability to utilize glucose efficiently leading to improved curdlan production yield

This study was focused on the enhanced fermentative production of curdlan by *Agrobacterium* sp. ATCC 31749 using MJDA for 168 h. Fermented broth samples were removed at 48, 96 and 168 h of fermentation and analyzed for curdlan production yield, cell mass, saponins, flavonoids and proteins concentrations. Yield of curdlan produced by *Agrobacterium* sp. ATCC 31749 in MJDA media (with 5, 10 and 15% JDA) were much higher than in CK (control) medium. There was no significant difference (p < 0.05) in curdlan production in each of the medium between 96 and 168 h of fermentation. Maximum utilization of sucrose and curdlan production occurred at 168 h. The maximum sucrose consumption by *Agrobacterium* sp. ATCC 31749 was 80.6% and was achieved in the 15% MJDA medium with 28.2 g/l curdlan production yield, whiles the highest amount of curdlan (40.2 g/l) was produced in 10% MJDA medium with 73.2% sucrose consumption followed by 5% MJDA with 34.9 g/l curdlan production yield and 68.9% sucrose consumption (Fig. 1a–c). CK medium produced 21.1 g/l of curdlan with 63.9% sucrose consumption. When the yield of curdlan was expressed in terms of g-curdlan-produced/g-sucrose-consumed, the following data was obtained: 1.0 g-curdlan/1.9 g-sucrose (5% MJDA), 1.0 g-curdlan/1.8 g-sucrose (10% MJDA), 1.0 g-curdlan/2.9 g-sucrose (15% MJDA) and 1.0 g-curdlan/3.0 g-sucrose (control). These values give an intuition that the mass of sucrose required to produce 1.0 g curdlan in the control medium is high than the mass of sucrose needed to produce 1.0 g of curdlan in all MJDA media.

**Fig. 2:**
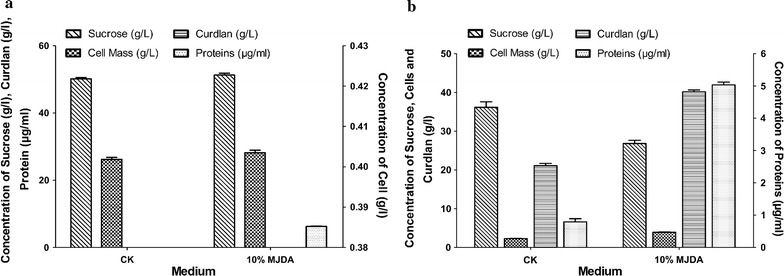
**a** Contents of CK and 10% MJDA medium at 0 h (before fermentation). CK medium contained only sucrose and cell mass. 10% MJDA contained sucrose, cell mass and proteins as well as flavonoids and saponins (not shown here). MJDA medium is enriched with nutrients essential for bacteria activity. **b** Contents of CK and 10% MJDA medium after 168 h of fermentation. The yield of curdlan in 10% MJDA is higher than in CK

All the MJDA culture media contained flavonoids, saponins and proteins but in different concentrations since different volumes of JDA were used to prepare different MJDA media. MJDA media containing higher percentages of JDA, contain higher amounts of proteins (nitrogen). There was no significant difference (p < 0.05) in total flavonoids, saponins and proteins contents between all AJ media before fermentation and after 168 h of fermentation (Fig. 2a, b).

### Curdlan produced from MJDA contains saponins and flavonoids

**Fig. 3:**
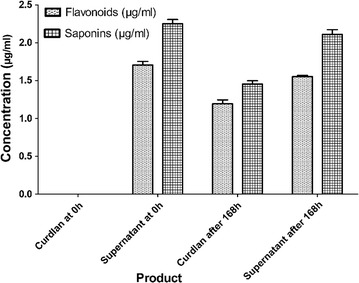
Flavonoids and saponins concentrations in curdlan and supernatant. At 0 h, only supernatant contains flavonoids and saponins since no curdlan was produced at 0 h. 10% MJDA curdlan contained 1.2 and 1.5 µg/ml of flavonoids and saponins respectively whiles the supernatant of 10% MJDA contained 1.6 µg/ml of flavonoids and 2.1 µg/ml of saponins after 168 h of fermentation

Flavonoids and saponins were found to be present in curdlan produce by all MJDA media but not in CK. Curdlan produced by 10% MJDA contained 1.2 and 1.5 µg/ml of flavonoids and saponins respectively. The supernatant of 10% MJDA contained 1.5 µg/ml of flavonoids and 2.1 µg/ml of saponins after extraction of curdlan after 168 h of fermentation (Fig. 3). There was no significant difference (p < 0.05) in total flavonoids, saponins in the curdlan and supernatant since they were in the same solution (medium) from which they were produced.

## Discussion

Asparagus spear is rich in minerals and vitamins [36]. Vitamins and minerals are necessary for bacteria activity and exopolysaccharide production. The rich minerals and vitamins content of all MJDA media improved the ability of *Agrobacterium* sp. ATCC 31749 to utilise more sucrose and at a faster rate than the control medium. This enhanced cell growth and metabolic activities of *Agrobacterium* sp. ATCC 31749 cells, leading to production of higher concentration of cells which resulted in yielding higher mass of curdlan produced in MJDA compared to CK from 48 to 168 h. The MJDA enhanced the cell’s ability to utilise sucrose to increase the production of curdlan up to 1.9-fold resulting in 90.4% higher in curdlan production in 10% MJDA medium compared to the control. The rate of curdlan production was also higher, 0.24 g/l/h, in 10% MJDA, compared to the 0.13 g/l/h in the control. For the 5% MJDA, less amount of curdlan was produced since the amount of vitamins and minerals was minimal as compared to the 10% MJDA. Therefore, the 10% MJDA medium contained the adequate amount of vitamins and minerals as well as nitrogen source for effective and higher yield of curdlan.

Asparagus also contains 52.9% carbohydrates which are the main carbon source for bacterial exopolysaccharide production [37]. This extra carbon source improved curdlan production by supplementing the amount of sucrose in MJDA media since sucrose concentration is crucial for curdlan synthesis by *Agrobacterium* sp. ATCC 31749. Increasing percentages of JDA in MJDA media was proportional to the amount cell mass produced, but was not proportional to the curdlan produced, since after 168 h of fermentation, 5% MJDA medium with 3.5 g/l cell mass produced 34.9 g/l curdlan, 10% MJDA medium with cell mass of 3.9 g/l produced 40.2 g/l of curdlan but 15% MJDA medium with 4.2 g/l cell mass produced 28.2 g/l of curdlan. Curdlan production is effective at nitrogen starvation conditions. Therefore, the lower amount of curdlan produced in 15% MJDA (and the other higher percentages of MJDA) is due to the excess nitrogen (from proteins in JDA) available in the 15% MJDA medium and this cause the observed lower amount of curdlan production in 15% MJDA medium. The yield of curdlan, when expressed in terms of sucrose consumed by cells, gave: 1.0 g-curdlan/1.9 g-sucrose (5% MJDA), 1.0 g-curdlan/1.8 g-sucrose (10% MJDA), 1.0 g-curdlan/2.9 g-sucrose (15% MJDA) and 1.0 g-curdlan/3.0 g-sucrose (control). This data denotes that the mass of sucrose required to produce 1.0 g curdlan in the control medium is higher than the mass of sucrose needed to produce 1.0 g of curdlan in all MJDA media. That is, the mass of sucrose needed to produce 1.0 g curdlan by *Agrobacterium* sp. ATCC 31749 in CK is 1.7-fold more than in 10% MJDA. This confirms that there is an improvement in curdlan yield.

Asparagus is known to contain saponins, flavonoids, and many other valuable constituents, such as protein, fat, sugar, amino acids, vitamins, and minerals [38]. Therefore, all the MJDA culture media contained flavonoids, saponins and proteins but in different concentrations since different volumes of JDA were used to prepare different MJDA media. MJDA media containing higher percentages of JDA, contain higher amounts of proteins (nitrogen). Higher amount of proteins increased cell mass at the growth phase thereby promoting curdlan production, but inhibited curdlan production at the production phase due to excess nitrogen (proteins) in MJDA (Fig. 1a). This is because curdlan production by *Agrobacterium* sp. ATCC 31749 is stimulated by and effective under nitrogen depleted conditions [39]. Also, the saponins and flavonoids in MJDA media may have contributed to the observed lower amount of curdlan produced in higher concentrations of MJDA, because saponins and flavonoids hinder bacterial activity [40]. Asparagus is a good source of minerals and vitamins. Therefore, no minerals were added to the MJDA media, but they produced higher amounts of curdlan than the CK medium in which minerals were added. The MJDA media were richer in natural plant source of minerals and vitamins from Asparagus, hence they produced higher masses of curdlan than the CK which contained chemical source of minerals and vitamins. These vitamins and minerals already contained in the MJDA are necessary for bacteria activity and exopolysaccharide production and hence contributed to the improved production of curdlan by enhancing growth and metabolic activities of *Agrobacterium* sp. ATCC 31749.

Curdlan produced by all MJDA media were found to contain flavonoids and saponins. These flavonoids and saponins contained in the curdlan are from the JDA used to prepare the MJDA. Flavonoids and saponins have wide range of pharmacological properties and these dietary products occur naturally in fruit, vegetables and tea. Pharmacological effects of flavonoids include antioxidative and antimutagenic [41, 42], free radical scavenging capacity, coronary heart disease prevention [43], hepato-protective, antiinflammatory, antiallergic, antidiabetic, gastro-protective, antiviral, and antineoplastic activities [44, 45]. Saponins possess anticarcinogenic, immunostimulatory and hypocholesterolemic activities [46, 47]. Hence curdlan produced in MJDA (MJDA-curdlan) will possess all these pharmacological properties due to the presence of flavonoids and saponins. Curdlan is extensively used in food and pharmaceutical industries as supplements, additives, stabilizers and as drug vehicle respectively. In food industries, when food products are supplemented with MJDA-curdlan, consumers will benefit from the health benefits of flavonoids and saponins such as antibacterial, anticarcinogenic and immunostimulatory. Use of MJDA-curdlan in processing foods such as noodles will help improve health of consumers and also improve the viability and longevity of the food products by preventing decay of curdlan (indirectly preventing decay of the food products) due to the presence of saponins and flavonoids. The flavonoids and saponins contained in the MJDA media supernatant (Fig. 3) after extraction of curdlan, can also be concentrated and extracted for use as supplements to other foods or added to the curdlan, thereby reducing the cost of buying and applying these dietary products in food and pharmaceutical industries.

The ability of *Agrobacterium* sp. ATCC 31749 to use MJDA media is a necessary step toward using cheap and abundant Asparagus shoot underground discarded part for value-added products, such as curdlan, thereby reducing environmental waste.

## Conclusion

The MJDA enhanced the cell’s ability to use sucrose and increased the production of curdlan up 90.4% higher in 10% MJDA media than the control. The best percentage of JDA in MJDA medium for highest production of curdlan is 5–10% MJDA. The results shown here strongly reveal that MJDA can be used to supplement curdlan fermentation media to increase curdlan production with safe and very low cost of production, thereby reducing environmental waste posed by the abundant discarded bottom parts of green Asparagus spear during Asparagus production. Curdlan produced by MJDA contains saponins and flavonoids as additives which has wide range of health benefits. Saponins and flavonoids in fermented broth can be concentrated and extracted for use as food and drug additives.

## Abbreviations

JDA: juice of discarded underground bottom part of Asparagus spear
MJDA: medium containing juice of discarded underground bottom part of Asparagus spear
ATCC: American Type Culture Collection
CFM: curdlan fermentation medium
LB: Luria-Bertani CK: control medium
